# Therapeutic Potential of Plant Phenolic Acids in the Treatment of Cancer

**DOI:** 10.3390/biom10020221

**Published:** 2020-02-03

**Authors:** Mariam Abotaleb, Alena Liskova, Peter Kubatka, Dietrich Büsselberg

**Affiliations:** 1 Department of Physiology and Biophysics, Weill Cornell Medicine-Qatar, Education City, Qatar Foundation, Doha, P.O. Box 24144, Qatar; mariam.abotaleb@aucegypt.edu; 2 Department of Obstetrics and Gynecology, Jessenius Faculty of Medicine, Comenius University in Bratislava, 036 01 Martin, Slovakia; alenka.liskova@gmail.com; 3 Department of Medical Biology, Jessenius Faculty of Medicine, Comenius University in Bratislava, 036 01 Martin, Slovakia; peter.kubatka@uniba.sk

**Keywords:** phenolics, cancer, proliferation, apoptosis, metastasis, benzoic acids, cinnamic acids

## Abstract

Globally, cancer is the second leading cause of death. Different conventional approaches to treat cancer include chemotherapy or radiotherapy. However, these are usually associated with various deleterious effects and numerous disadvantages in clinical practice. In addition, there are increasing concerns about drug resistance. In the continuous search for safer and more effective treatments, plant-derived natural compounds are of major interest. Plant phenolics are secondary metabolites that have gained importance as potential anti-cancer compounds. Phenolics display a great prospective as cytotoxic anti-cancer agents promoting apoptosis, reducing proliferation, and targeting various aspects of cancer (angiogenesis, growth and differentiation, and metastasis). Phenolic acids are a subclass of plant phenolics, furtherly divided into benzoic and cinnamic acids, that are associated with potent anticancer abilities in various in vitro and in vivo studies. Moreover, the therapeutic activities of phenolic acids are reinforced by their role as epigenetic regulators as well as supporters of adverse events or resistance associated with conventional anticancer therapy. Encapsulation of phyto-substances into nanocarrier systems is a challenging aspect concerning the efficiency of natural substances used in cancer treatment. A summary of phenolic acids and their effectiveness as well as phenolic-associated advances in cancer treatment will be discussed in this review.

## 1. Introduction

**Figure 1:**
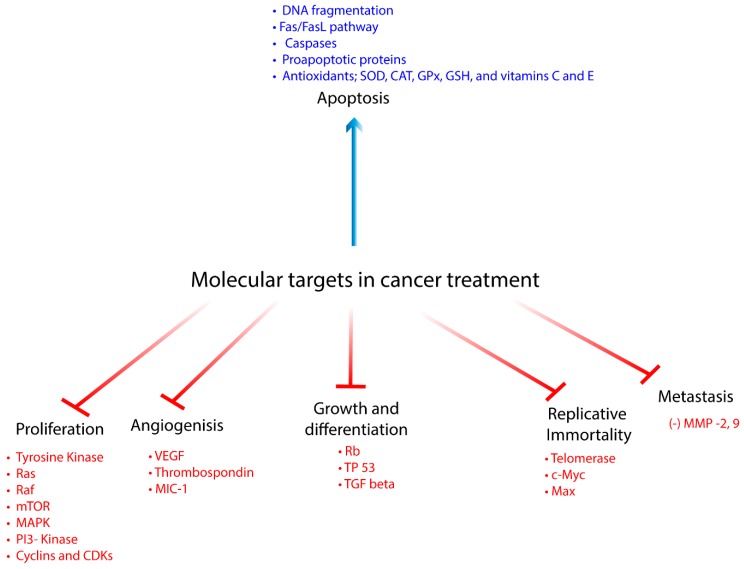
Molecular pathways targeted in cancer treatment.

Cancer is defined as an irreversible impairment in cellular homeostasis. It is a multifactorial, heterogeneous metabolic disease. This impairment may be associated with internal sources such as loss/ reduced cellular functions; apoptosis, oxidative stress, mutations and hypoxia, or emerging from external origins listed as prolonged exposure to radiation, ultraviolet rays, pollution, in addition to smoking, and stress. Cancer progression is marked by six main hallmarks: (1) uncontrolled cell growth and differentiation, (2) replicative immortality, (3) promotion of angiogenesis, (4) increased proliferative signaling, (5) resistance in cell death and finally (6) metastatic invasion. Thus, targeting these molecular pathways attempting to repair the disturbed balance is required to control and treat of cancer (Figure 1) [1].

**Figure 2:**
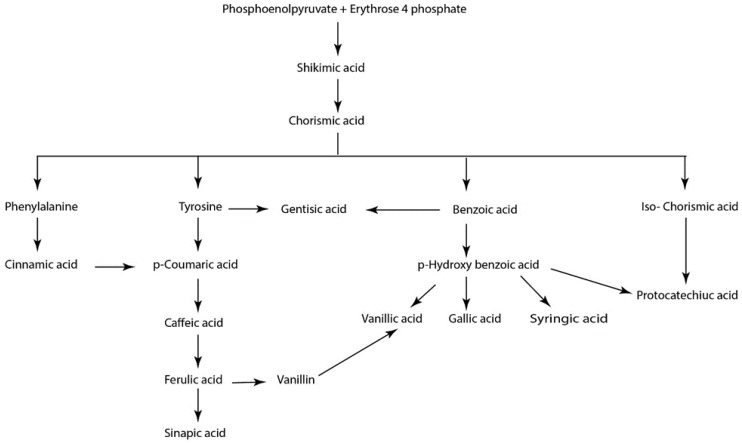
Biosynthesis of phenolic acids in plants via the shikimic acid pathway. Adapted from [3].

Phenolics are vital constituents in plants frequently found in fruits, vegetables, cereals, and legumes. In plants they function in wide range of processes such as pigmentation, growth, reproduction and resistance to pathogens or predators. They are classified into five main classes: coumarins, flavonoids, phenolic acids, stilbenes, and tannins [2,3]. Phenolics are secondary metabolites mainly produced in plants from shikimic acid via the phenylpropanoid pathway (Figure 2). They are also produced as a result of the breakdown of lignin and cell wall polymers in vascular plants and as by-products of the monolignol pathway [2,3].

**Figure 3:**
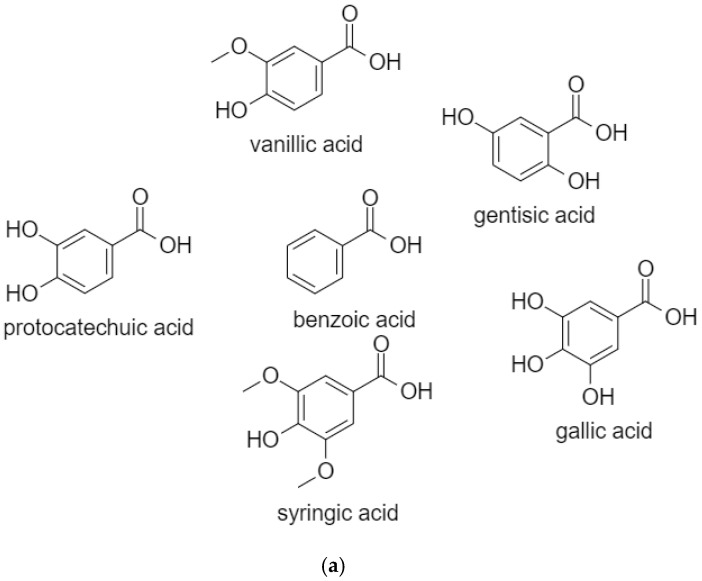

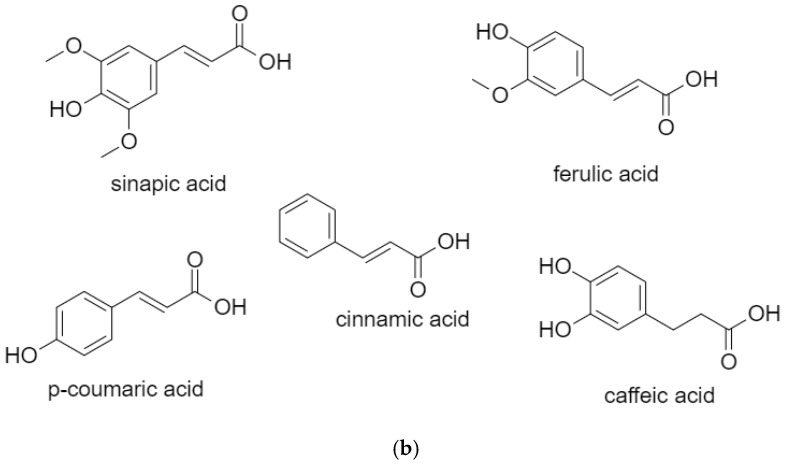
(**a**) Benzoic acids: vanillic, gentisic, protocatechuic, gallic, and syringic acid; (**b**) Cinnamic acids: caffeic, ferulic, *p*-coumaric, and sinapic acid.

Phenolic acids are additionally classified as benzoic acids (Figure 3a) or cinnamic acids (Figure 3b). The direct biosynthetic route of benzoic acids is unclear; however, they are usually produced from cinnamic acid and its derivatives. Frequently found examples are: *p*-hydroxybenzoic acids including vanillic, protocatechuic, syringic, and gallic acid, in addition to gentisic acid, derived from catabolism of tyrosine [3,4], while cinnamic acids are *ortho*-oxygenated then subsequently methylenated, leading to the formation of the majority of hydroxycinnamic acids such as ferulic, *p*-coumaric, caffeic, and sinapic acid. Phenolic acids are found in free forms or conjugated with esters, ethers and a variety of other molecules (simple sugars, organic acids and plant polymers) [2,3,5,6]. Key structural motifs required for the anticancer activity of phenolic compounds include the aromatic ring, unsaturated substituted chains and the number and position of free hydroxyl groups [3]. This review aims to concentrate on the potential of phenolic acids as cytotoxic agents in terms of mechanism of action and therapeutics.

## 2. Therapeutic Effects of Phenolics in Preclinical Cancer Research

Phenolics are popular for their potency as medicinal compounds in treatment of various diseases such as diabetes, cardiovascular and neurodegenerative diseases, as well as cancer. Their antidiabetic property is mediated through the modulation of glucose metabolism [2]. Their potency as anticancer compounds is primarily attributed to their antioxidant activity; being strong radical scavengers, metal chelators, modifiers of endogenous defense mechanisms as superoxide dismutase (SOD), catalase (CAT), glutathione peroxidases (GPx), enhancers of glutathione (GSH) redox status, and regulators of diverse proteins and transcriptional factors such as nuclear factor erythroid related factor (NRF2) [2,7]. Moreover, their anticarcinogenic effects is associated with their ability to inhibit cell proliferation (extracellular signal-regulated kinase (Erk)1/2, D-type cyclins, and cyclin-dependent kinases (CDKs)), angiogenic factors (vascular endothelial growth factor (VEGF) and MIC-1), oncogenic signaling cascades (phosphoinositide 3-kinase (PI3K) and protein kinase B (Akt)), inducing apoptosis, and preventing cellular migration and metastasis [2,5]. 

### 2.1. BenzoicAacids 

#### 2.1.1. Vanillic Acid 

Vanillic acid (4-hydroxy-3-methoxybenzoic acid, VA) is produced from ferulic acid in a reaction that involves vanillin as an intermediate [8,9]. It is a major active compound isolated from *Angelica sinensis* and green tea [8]. VA acts as a chemo-protectant and contributes in the prevention of benzo(a)pyrene-induced lung cancer in Swiss albino mice was indicated by its free-radical scavenging antioxidant activity [10]. VA also demonstrated antioxidant and anti-lipid peroxidative abilities in the DMBA-induced hamster buccal pouch carcinogenesis. Interestingly, administration of VA led to the restoration of levels of lipid peroxidation by-products and abnormalities in antioxidative status which were found to be altered after administration of DMBA alone [11]. In cancer treatment; the potential anticancer effectiveness of whole plant or isolated plant extracts containing several natural compounds as well was tested. Interestingly, transformed root extract of *Leonurus sibiricus* is rich in various polyphenolic compounds including VA as well as gentisic acid,4-hydroxybenzoic acid, 1,3-dicaffeoylquinic acid, and α-resorcylic acid, demonstrated an ability to inhibit proliferation and to induce apoptosis in glioma cells with these effects mediated via generation of reactive oxygen species (ROS), loss of mitochondrial membrane potential, enhancement of S and G2/M cell cycle phases, and changes in mRNA levels of apoptotic factors including Bax, Bcl-2, p53, Caspase-3, -8 and -9 [12]. In HCT116 colorectal cancer cells, isolated VA inhibited angiogenesis and proliferation and led to cell cycle arrest at G1 phase by inhibiting the expression of hypoxia inducing factor (HIF-1) protein synthesis in a dose dependent manner (30 µM) without affecting its degradation or its mRNA expression. Thus, this inhibitory effect depends on the inhibition of mTOR/p70S6K/4E-BP1 and Raf/MEK/ERK pathways [8]. The in vitro antioxidant capacity of VA was demonstrated through the reduced DNA damage, induced by H_2_O_2_ in human lymphocytes at concentrations of 0.17–67.2 μg/mL [9]. While, in rat models of N-methyl-N’-nitro-N-nitrosoguanidine (MNNG)-induced endometrial carcinoma treatment (100 mg/kg body weight) showed high antioxidant potency, their action involved increasing the levels of cellular antioxidants; SOD, CAT, GPx, GSH, and vitamins C and E in plasma and uterus, as well as reducing oxidative damage by decreasing the levels of thiobarbituric acid reactive substances (TBARS), lipid hydroperoxides (LOOH) and cytochrome p450. The same study additionally, demonstrated the reduction of cancer progression and metastasis by down regulation of the expression of oncogene cyclin D1 and matrix metalloproteinases (MMP) -2, -9 [13].

#### 2.1.2. Gentisic Acid

Gentisic acid (2,5-dihydroxybenzoic acid, GeA) is a biosynthetic derivative of salicylic acid and a byproduct of tyrosine catabolism found in citric fruits, grapes, artichokes, sesame, and olives. It has antioxidant and anti-inflammatory effects and is also used in the treatment of cardiovascular diseases [14,15]. The potent antioxidant effects of GeA could be direct as it is a free radical scavenging molecule or indirect by acting as an agonist of NRF2 which regulates the synthesis of antioxidant molecules [14]. The anticancer effectiveness of *Vaccinium myrtillus whole* extract on HCT-116 cells, demonstrated to be an effective antioxidant. It is worth mentioning that GeA is considered to be one of the most abundant polyphenols found in this extract along with quercetin and kaempferol [16].

Importantly, the administration of GeA at clinically available doses led to the blockage of growth, DNA synthesis, and colony formation in C6 glioma in vitro. Considering in vivo model, GeA enhanced the survival of Ehrlich breast ascites carcinoma-bearing mice. Regarding the growth of Ehrlich solid tumors (EST) affected by GeA alone or by sodium selenite, GeA reduced their growth and did not modify antineoplastic effects of selenium which initially decreased EST. However, GeA led to the blockage of the selenite’s tumor stimulation at later stage. Lastly, GeA attenuated side effects of doxorubicin including myofibrillary and endothelial damage as well as hyalinization necrosis [17]. Additionally, GeA has an indirect role in controlling brain glioblastoma as it blocks the OAT3 and solute carrier-22A8 responsible for brain efflux of anticancer drugs, leading to accumulation of chemotherapeutic agents in brain hence, reducing the tumor growth. Moreover, GeA and its isomers regulate cell cycle progression by blocking CDK 1 enzymatic activity [14]. 

#### 2.1.3. Protocatechuic Acid

Protocatechuic acid (3,4-dihydroxybenzoic acid, PCA) is found in plum, star anise, melissa, rosemary, cinnamon, sudan mallow, St. John’s wort, berries, cauliflower, and lentils. It has a wide range of biological and pharmacological activities including antioxidant, antibacterial, anticancer, antiulcer, antidiabetic, antifibrotic, antiviral, anti-inflammatory, analgesic, cardiac, antiaging, hepatoprotective, neurological, and nephroprotective efficacy [5]. In cancer, PCA promoted cell death in HepG2 cell lines by stimulating the JNK and p38-MAPK [18,19]. Moreover, PCA exerted apoptotic and antiproliferative effects in HL-60 leukemia and human gastric adenocarcinoma (AGS) cells which were manifested by increased DNA fragmentation and Bax expression, reduced Bcl-2 protein expression, induction of RB phosphorylation, and activation of Fas/FasL pathway [19]. PCA also induced mitochondrial apoptotic cell death in PC12 cells through loss of mitochondrial membrane potential and induction of ROS [19]. In addition, treatment of three ovarian cell lines (SKOV-3, OVCAR-3, and A2780) with PCA showed a significant reduction in viability and colony formation, the mechanism was illustrated to be through the induction of apoptosis, autophagy and cell cycle arrest at G2/M phase, concluded by the activation of Poly-(ADP-Ribose)-Polymerase (PARP), upregulation of caspase-3 and Bax, as well as a downregulation of Bcl-2. PCA administration also led to upregulation of autophagy-related protein LC3-II and induction of GFP-LC3 puncta formation. An inhibitory efficacy of PCA on OVCAR-3 cells may be associated with increased glutathione levels and decreased intracellular ROS [20]. Furthermore, PCA was effective in penetrating cancer cells inducing lactate dehydrogenase leakage and disrupting the mitochondrial membrane potential by decreasing Na+/K+-ATPase activity in various cell lines including MCF-7, A549, HepG2, HeLa, and LNCaP cells. Additionally, antimetastatic potential of PCA in human gastric carcinoma AGS cells was mediated via the inhibition of MMP-2 secretion [21]. Moreover, PCA at 25 µM showed potent anti-angiogenic activities in in vitro study using HUVECs as it blocked cellular proliferation, migration, invasion and increased ROS generation thus inhibited VEGFR2-dependant Akt/MMP2 and ERK pathways [22]. In human gastric carcinoma AGS cells antimetastatic effect was mediated via the inhibition of MMP-2 secretion [21]. Additionally, the antimetastatic effects of PCA were evaluated in AGS cells used in wound healing model and Boyden chamber assay in vitro and in metastasis in vivo model of B16/F10 melanoma cells injected in mice. Essentially, antimigratory and antiinvasive abilities of PCA were found to be associated with decreased expression MMP-2 via the down-regulation of the Ras/Akt/ nuclear factor-kappa (NF- κB) pathway by targeting RhoB activation. Additionally, an inhibition of metastasis of B16/F10 melanoma cells to the liver as a result of PCA administration was also observed [23]. 

In cancer therapy the protective effect of PCA was also demonstrated in reducing nephrotoxicity in the cisplatin-treated rats in a dose dependent manner, such that PCA co-treatment at doses of 10 and20 mg/kg body weight resulted in remarkable improvement in the histological appearance and reduction in tubular cell damage, reduction of elevated levels of pro-caspase-3 induced by cisplatin in rat kidneys [24]. In nanomedicine and drug delivery it is associated with improvements in the therapy efficacy, reduction of side effects and prolonged bioavailability. Therefore, graphene oxide–polyethylene glycol (GO-PEG) nanocarrier system was designed for the PCA. Due to the overexpression of folate receptor on majority of cancer tissues, folic acid coating (FA) was employed in this nanocarrier to target cancer cells (GO-PEG-PCA-FA). Importantly, GO-PEG-PCA-FA nanocarrier system was demonstrated to be more effective anti-cancer agent when compared with free PCA as well as uncoated delivery system against colon cancer HT-29 and HEP-G2 cells. Moreover, in vitro release of PCA was found to be highly sustained and extended [25]. Similarly, the effectiveness of PCA intercalated in the nanocarrier zinc-aluminium-layered double hydroxide (PCA-ZnAl) system against diethylnitrosamine/phenobarbital-induced hepatocellular carcinoma was evaluated in BALB/c mice. Intercalated PCA is more thermally stable and its release from the carrier system is sustained and controlled. Actually, both PCA and doxorubicin remarkably reversed tumor marker expression as well as lung and kidney structures and weight gain. However, PCA-ZnAl group was associated with better or similar improvements in comparison with doxorubicin treatment or the administration of plain nanocomposites [26].

#### 2.1.4. Gallic Acid

Gallic acid (3,4,5-trihydroxybenzoic acid, GA) is extracted from chestnut green chicory, blackberry, raspberry, walnuts, chocolate, wine, green tea, and vinegar either in free form or as a part of hydrolysable tannins. It has strong anti-microbial, anti-inflammatory, and anticancer activities. Its anticancer activities are mainly exerted by preventing cellular proliferation, promotion and generation of reactive oxygen species (ROS) and cell cycle arrest in G2/M phase [5,27]. Aqueous extract from *Rhus verniciflua* (RVSE) upregulated p53 and p21 and thus induced apoptosis in sub-G1 phase in MCF-7 breast cancer cells. Importantly, high-performance liquid chromatography of RVSE revealed only the presence of GA, hence, an antiproliferative efficacy on MCF-7 cells may be attributed to the effects of GA [28].

At low concentrations (5 μM) GA selectively inhibited the growth and in vitro angiogenesis of two ovarian cancer cell lines OVCAR-3 and A2780/CP70 in a concentration-dependent manner. GA inhibited VEGF secretion through suppression of Akt phosphorylation and HIF-1α expression and promotion of PTEN expression [29]. In glioblastoma multiforme (GBM) T98G cell lines GA antiproliferative effect was associated with epigenetic alterations in miRNAs initiating apoptotic cell death at low concentrations [30]. Additionally, GA decreased IL-6 protein level resulting in suppressing pAkt signaling by blocking pSTAT3, pERK1/2, consequently leading to the reduction of the survival, proliferation, and invasion in PC3 cells [31]. While the epidermal growth factor receptor (EGFR)-dependent anti-proliferative and apoptotic effects of GA in malignant mesothelioma (SPC212) cells were demonstrated to be dually concentration- and time-dependent. Importantly, the activation and upregulation of ERK1/2, EGFR, Akt proteins and the expression of p21 gene along with the downregulation of Cyclin D and Bcl-2 genes were demonstrated by GA, in EGF-induced SPC212 cells leading to a transitory G1 arrest and triggering of mitochondrial and p38MAPK mediated apoptosis [32]. The proapoptotic effect of GA in DBTRG-05MG cells was shown by altering calcium ion homeostasis from the endoplasmic reticulum in a dose dependent manner, evoking mitochondrial apoptotic pathways involving ROS production [33]. Similarly in, cervical cancer cells HeLa and human umbilical vein endothelial cells (HUVEC), GA led to apoptotic cell death via the induction of ROS and GSH accompanied by the loss of mitochondrial membrane potential [34]. The ROS-dependent pro-apoptotic effects of GA were also demonstrated in declined viability of HCT-15 colon cancer or LNCaP prostate cancer cells [27]. The beneficial effects of GA in triple-negative breast cancer (TNBC) were suggested by the study that revealed an ability of GA to induce G1 phase arrest and apoptosis via p38 mitogen-activated protein kinase/p21/p27 axis in MDA-MB-231 cells [35]. Moreover, GA was associated with p53-involved upregulation of Fas, FasL, and DR5 and apoptosis in AGS cells [36]. In addition, epigenome-protecting abilities of GA were also observed in tobacco-associated cancers. GA reduced nuclear and cytoplasmatic DNA methyltransferases in H1299 cells [37]. GA was also found to be a potent inhibitor of HDAC8 and class IIa/b HDAC activity [38].In addition, GA demonstrated anti-invasive effects in human nasopharyngeal carcinoma cells NPC-BM1 through the inhibition of p38 MAPK signaling pathway, this was due to suppressed transcription of MMP-1 by down-regulation of Ets1 and c-Jun, c-fos of the AP-1 [39]. In case of AGS cells, GA up-regulated of RhoB and the down-regulated Akt/small GTPase signals and NF- κB activity inhibited cell migration [40]. In addition to its role in suppressing the invasion and migration of PC-3 prostate cancer cells through down-regulation of MMP-2 and MMP-9 [27]. Furthermore, an ability of GA to inhibit viability of lung carcinoma A549 cells was related to the GA upregulating voltage dependent anion-selective channel protein 1 [41]. In addition, the anticancer and auxiliary effects of GA were observed in NSCLC A549 cells treated with cisplatin. GA inhibited the proliferation and induced the apoptosis in a dual manner, which was associated with upregulated Bax and downregulated Bcl-2 and modulated the JAK/STAT3 signaling [42].

In therapeutics a post-fermentation oolong tea extract (PFOTE) GA-enriched using *Aspergillus sojae* was associated with further enhanced demethylation abilities. PFOTE also increased sensitivity to cisplatin and showed stronger antiproliferative abilities when compared with oolong tea extract [37]. Furthermore, an ability of GA to potentiate side effects of conventional chemotherapeutic drugs was evaluated in HeLa cervical cancer cells. Interestingly, the combination of GA and Paclitaxel was associated with lower side effects and thus may represent a replacement of Paclitaxel/Carboplatin combination which is currently one of the most commonly used drugs in the therapy of cervical cancer [43]. Similarly, the potential of GA at 50 and 100 µM as a co-adjuvant to Paclitaxel was demonstrated by its ability to sensitize Paclitaxel-resistant ovarian cancer cells (A2780AD) via inactivation of ERK which was associated with an increase in ROS [44]. Additionally, in multidrug resistant small cell lung cancer SCLC H446, GA showed promotive effects to cisplatin, as shown by it changed morphology, inhibited the growth and induced apoptosis generated of ROS, disruption of MMP, downregulation of XIAP expression, and upregulation of Bax, Apaf-1, DIABLO and p53 expression [45]. Similarly, as in case of GeA, iron oxide magnetite nanoparticles coated with polyethylene glycol and loaded with GA (Fe₃O₄-PEG-GA) were found to be more effective against human lung A549, breast MCF-7, and colon HT-29 cells when compared with free GA [46]. Moreover, encapsulation of GA into PLGA-CS-PEG nanocomposite was also associated with increase in its bioavailability and antitumor efficacy in rats [47]. 

#### 2.1.5. Syringic Acid 

Syringic acid (4-hydroxy-3,5-dimethoxybenzoic acid, SyA) is an abundant phenolic compound present in dates, olives, pumpkin, grapes, spices, acai, red wine, palm and honey [5,7,48]. It has higher potency than *p*-hydroxybenzoic acid due to the free radical scavenging activity attributed to the presence of two methoxy moieties at positions 3 and 5 [7]. Apparently, non-melanoma skin cancer is related to excessive UV exposure. The study focusing on an association between SA and UVB-induced signaling and skin cancer revealed that chemo-preventive potential in vitro and in vivo was mediated mainly via an ability of SA to inhibit Nox/PTP-κ/EGFR axis. Specifically, the protective efficacy of SyA in human epidermal keratinocytes HaCaT cells is based on the ability to inhibit UVB-induced COX-2, MMP-1, prostaglandin E2 expression, activator protein-1 activity, phosphorylation of mitogen-activated protein kinases and Akt, EGFR as well as ROS formation. Moreover, SyA along with the administration of antioxidant N-acetyl-L-cysteine constrained the action of UVB-induced NADPH oxidase activity. *In vivo*, SyA pretreatment led to the dose-dependent suppression of UVB-induced skin cancer incidence in mice [48]. *Menyanthes trifoliate* root extracts containing terpenoids and polyphenols including SyA led to induction of apoptosis mediated via G2/M phase cell cycle arrest and alterations in the expression of Bax, Bcl-2, cas-3 nad p53 as well as the decrease in the mitochondrial membrane potential in IV glioma cells [49]. Additionally, Manuka honey (MH) is considered as the source of various phenolic compounds such as GA and SyA. MH treatment led to the cell cycle arrest at S phase in HCT-116 cells and at G2/M phase in the LoVo cells. Moreover, MH caused promoted apoptosis via increase in p53, cleaved-PARP, caspase-3, activation of extrinsic and intrinsic apoptotic pathways as well as suppression of p-Akt and increase in expression of p-p38MAPK and endoplasmic stress markers [50]. Similarly, isolated SyA induced apoptosis via mitochondrial pathway by elevating the expression of caspases 3 and 9, cytochrome c, Apaf-1, Bax, and p53 in contrast to the downregulation of Bcl-2 gene expression and caused liberation of ROS in HepG2 cells [51].

SyA also showed high potency in treatment of DMBA-induced hamster buccal pouch carcinogenesis (HBPC) in a dose dependent manner. Results showed that anti-lipid peroxidative, antioxidant, anti-cell proliferative, and apoptosis-inducing properties of SyA were mediated by down-regulation of TBARS, LOOH, enzymatic (SOD, CAT and Gpx) and non-enzymatic antioxidants (vitamin E and GSH) and reduction of the expression of PCNA, Cyclin D1, and mutant p53 [52]. Moreover, SyA was found to repress cell surface glycoconjugate abnormalities induced by 7,12-dimethylbenz(a)anthracene and to restore expression of cytokeratin in the plasma and buccal mucosa of golden Syrian hamster buccal pouch carcinogenesis. Results suggested that SyA possess membrane stabilizing effects during neoplastic changes with its efficacy mediated probably via inhibition of the abnormal GPs separation and regulation of glycosyltransferase activity [53]. It also exhibits antimitogenic and chemo sensitizing activity in human colorectal cancer SW1116 and SW837 cell lines by time-dependent induction of cell-cycle arrest at S/G2-M, G1/G2-M and S/G2-M phase and apoptosis, inhibition of cell migration and NF- κB, and DNA binding. Cell cycle arrest and apoptosis are elucidated by increased S-phase, downregulated cell cycle proteins CDK4, CDK6 and cyclins B, C, E1, H and upregulated p19, p21^Cip1/Waf1^ and p27^kip1^ expression. Moreover, apoptosis is also induced by the upregulation of expression of the proapoptotic genes (Bax, Bak, Bad, Bid, Bim, Apaf1, AIF Smac, caspases-2, 3, 6, 7, 8 and 9) [54]. 

### 2.2. Cinnamic Acids

Hydroxycinnamic acids are synthesized in various plants and fruits such as coffee beans, tea, berries, tomatoes, citrus, grapes, spinach, beetroots, artichokes, potatoes, and cereals. They display anticancer, antiatherogenic, antimalarial, antifungal, antimicrobial, and antioxidant activities [2]. Cinnamic acids esters are considered to be the most potent class when compared to methoxylated or hydroxylated forms [3]. 

#### 2.2.1. Caffeic Acid

Caffeic acid (3,4-dihydroxycinnamic acid, CA) is found as ester form in wheat, quinoa, triticale, barley, corn, oat, rye, rice, thyme, oregano millet, sage, and sorghum [2,5]. CA and its derivatives are well known for their antibacterial, anti-mutagenic, anti-inflammatory, and anti-carcinogenic properties, which could be linked to its antioxidant activity [55]. Their antioxidant effects are mediated by modulating signaling pathways such as NF- κB, MAPK, and Akt. Moreover, they induce cell cycle arrest and enhance apoptosis in neck, tongue, and mouth carcinomas [5]. In Ht-29 cell lines, both CA and 5-caffeoylquinic acid reduced cell viability via promotion of specific cell cycle alterations (increased cellular population at G0/G1, decrease in G2/M cells) and induced apoptosis in a time- and dose-dependent manner [3,56]. Interestingly, the antioxidant activity of CA could be related to its iron-chelating property through the formation of iron-CA complexes inhibiting Fenton-induced oxidative damage by preventing the formation of free hydroxyl radicals [55]. CA was also noted for its antioxidant effects by suppressing the production of ROS -SOD-, and prevention of cancer progression and migration by decreasing cell adhesion by reduced attachment to extracellular matrix (ECM) in human lung A549 and colon adenocarcinoma HT29-D4 cells [3,57]. Moreover, an evaluation of CA antitumor efficacy was performed on human cutaneous melanoma SK-Mel-28 cell line. Interestingly, CA exhibited an important role in the prevention of tumor progression via decrease in cell viability and induction of apoptosis. Moreover, CA treatment led to the cell cycle modulation, inhibition of colony formation, and changes in the expression of caspases [58]. Importantly, CA attenuated cancer stem cells-like properties via inhibition of TGFβ-SMAD2 signaling pathway mediated by microRNA-148a in vitro as well as in vivo mouse models [59]. Generally speaking, caffeic acid phenethyl ester (CAPE) is a component of honeybee propolis. Both CA and CAPE are considered to possess cell cycle inhibitory and proapoptotic properties in cancer cells [60]. Moreover, CAPE induced apoptosis in human multiple myeloma cells through oxidative stress with these effects mediated via activation of caspase-3 and PARP cleavage and decrease in intracellular antioxidant level [61]. Additionally, CAPE inhibited cancer progression through inactivation of NF-κB signaling in ovarian SKOV-3 cells [62] and attenuated proliferation and invasion of nasopharyngeal cancer cells via upregulation of NDRG1 expression through MAPK pathway and inhibition of STAT3 phosphorylation [63]. Actually, comparison of anticancer effects of CA and CAPE revealed dose and time-dependent ability of CAPE to be more potent in treating breast cancer cells with the mechanisms of its action mediated by inducing apoptosis, cell cycle arrest in MDA-MB-231 cells [60] and reducing the migration of MCF-7 [64]. Additionally, proapoptotic and anti-metastatic effects of pineapple vinegar (PV) were evaluated in mouse mammary gland cells in vitro and *in vivo*. PV rich in GA and CA was prepared in the process of double fermentation. Interestingly, 70 % of cell population underwent apoptosis and 30 % inhibited wound closure of 4T1 cells in response to the administration of PV. Therefore, anti-cancer efficacy of pineapple vinegar may be associated with the presence of phenolic acids, especially GA and CA in high content [65]. Moreover, methanol extracts *of Anchusa azurea* Mill, with CA as one of the four most abundant compounds, was found to induce programmed cell death in cancer cell line via activation of capsase-3/7 and -9 as well as modulation of cytoskeleton dynamics [66]. Interestingly, when combined with cisplatin; CA increased its therapeutic potential, the combinatory treatment led to the inhibition of cell growth of HeLa and CaSki cell lines which may be explained by the synergistic growth inhibition. Moreover, the combination of CA and cisplatin was also associated with increase in the expression of caspase-3, -7 and -9 [67].

#### 2.2.2. Ferulic Acid 

Ferulic acid (4-hydroxy-3-methoxycinnamic acid, FA) is one of the most abundant phenolic acids in plants. It is a byproduct derived from metabolizing phenylalanine and tyrosine found in wheat, buckwheat, rice, corn, oats, rye, orange, corn, herbs, spices, sorghum, millet, quinoa, and barley [2]. A wide range of therapeutic activities of FA is demonstrated in several diseases including diabetes, neurodegenerative, cancer and cardiovascular diseases [27]. Still, FA which is known antioxidant at lower concentrations, may act as a pro-oxidant leading to oxidative DNA breakage and ROS generation at higher concentrations (50 µM) or in the presence of chelator metal ions such as copper (25 µM) [27,68]. In prostate cancer PC-3 and LNCaP cell lines, FA inhibited cell proliferation, invasion and induced apoptosis at 300 μM and 500 μM, respectively [27,69]. Moreover, FA showed cytotoxic effects in the colorectal cancer Caco-2 cell line by elongating S/G2 phase and reducing G1 phase. Additionally, FA also exerted antioxidant effects by suppressing the production of superoxide anion and preventing cancer migration by reducing cell adhesion in adenocarcinoma lung A549 and colon HT29-D4 [3,57]. FA also showed protective effects to DNA damage induced by H_2_O_2_ and UV at 2 μg/mL [70]. Moreover, FA demonstrated ex vivo and in vivo inhibition of endothelial cell tube formation, proliferation and migration in response to basic fibroblast growth factor 1 (FGF-1). The suppression of melanoma growth and angiogenesis was carried out through FGFR1-mediated PI3K-Akt signaling pathway [71]. In addition, the anticancer efficacy of FA occurs by affecting colony formation, cell cycle, apoptotic and invasive and behavior of MIA PaCa-2 cells by increasing in the expression of Bax, p53, PTEN, caspases-3 and -9 and associated with decrease in the expression of CDK 4/6, cyclin D1 and Bcl-2 [72]. Furthermore, FA inhibited proliferation and induced apoptosis in 143B and MG63 osteosarcoma cells dose-dependently leading to G0/G1 phase arrest and apoptosis by down-regulating the expression of CDK 2, 4 and 6, upregulating Bax, downregulating Bcl-2, subsequently enhancing caspase-3 activity. It also inhibited PI3K/Akt activation in both cell lines [73,74]. FA role in suppressing metastasis was regulated by the reversal of epithelial-mesenchymal transition in vitro in MDA-MB-231 moreover, the inhibition of cell invasion through reducing MMP-9 mRNA expression [75]. 

In addition, FA suppressed invasion, migration, and colony formation leading to cell cycle arrest, apoptosis, invasion, migration, and colony formation in TT human thyroid cancer cell line as was demonstrated by decrease in expression of novel gene URG4/URGCP, CCND1, CDK4, and 6, Bcl-2, MMP2, and MMP9 and significant increase in the expression of p53, PARP, PUMA, NOXA, Bax, Bid, caspases-3 and -9 [76]. Moreover, the derivative of FA; FXS-3 inhibited metastasis and proliferation in human lung cancer A549 cells. Specifically, the administration of FXS-3 led to the apoptosis, G0/G1 arrest, increase in Bax/Bcl-2 ratio, MMP-2 inhibition and regulation of ERK/p38, JNK, AKT/mTOR and MEK/ERK signaling. Next, FXS-3 also suppressed metastasis and proliferation in A549 xenograft-bearing mouse and tail vein injection of A549 cells induced pulmonary tumor metastasis model. These results suggested FXS-3 to be a promising anticancer agent [77]. Furthermore, FA and 4-vinylguaiacol inhibited EGF-induced proliferation of breast cancer cells in vitro as well as synthesis of new DNA; therefore, it may potentially represent a structure for small molecule agents targeting EGFR [78].

In therapeutics the modulatory effect of FA on resistant ChR8-5 cells and tumor xenografts was mediated by FA synergistically enhancing doxorubicin-induced apoptotic signaling in the drug resistant cells. Also, NF-κB translocation was associated with the modulation of PI3K/Akt/signaling pathway [79]. It has limited applicability owing to its poor solubility;, FA is therefore, encapsulated into cyclodextrin nano sponges improved its solubility and in vitro cytotoxicity thus, representing a suitable delivery system with enhanced antiproliferative activity in MCF-7 and 4T1 breast cancer cell lines when compared with free FA [78]. Actually, the conjugation of nanoparticle with phytochemicals may represent a new approach in combinatorial chemotherapy as was demonstrated by the use of ZnO nanoparticle-FA conjugate in Huh-7, hepG2 cells and diethylnitrosamine-induced hepatocellular cancer on Wistar albino rat model [80]. Moreover, polyFE (chemically modified FE) loading doxorubicin nanoparticles maintained effective delivery of the drug in the acidic tumor microenvironment in vitro and reduced toxicity of free doxorubicin in vivo [81]. 

#### 2.2.3. *p*-Coumaric Acid

*p*-Coumaric acid (4-hydroxycinnamic acid, p-CA) is extracted from wheat, barley oat, corn, rye, quinoa, rice, millet, honey sorghum barley grains and buckwheat [2]. The inhibitory effect of p-CA was demonstrated in colon cancer cell lines HT 29 and HCT-15 by inducing mitochondrial mediated apoptosis through increasing the ROS levels, changing the mitochondrial membrane potential and inhibiting the cell cycle at sub G1 phase [3]. In *Rosa canina* extract p-CA demonstrated selective cytotoxic efficacy in human lung A549 and prostate PC-3 cells in comparison with normal fibroblast. The extract was also associated with arrest of the cell cycle at G1 phase and induction of apoptosis mediated through MMP reduction and increase in caspase activity [82].

Isolated p-CA demonstrated also chemo protectant effects in stomach cancers by reducing the formation of carcinogenic nitrosamines. In addition, p-CA exhibited excellent free radical scavenging and NF- κB modulatory activities [83]. Furthermore, p-CA administration led to the inhibition of glucose related protein 78, which is often deregulated in colon cancer, and activation of unfolded protein response-mediated apoptosis in in vitro and in vivo models of colon cancer. p-CA was also associated with a decrease in the expression of IL-6, COX-2, TNF-α, PGE2, p-p65 and p-IκBα and thus reduced inflammation [84]. The study evaluating protective efficacy of p-CA against 1,2 dimethylhydrazine (DMH)-induced colonic preneoplasia in rats demonstrated significant ability of p-CA to suppress DMH-associated preneoplastic lesion. Moreover, p-CA also exerted strong antioxidant response and detoxification mechanism and thus protected the colon against genotoxic insult with 100mg/kg body weight to be the most optimal dosage [85]. Similarly, p-CA supplementation in DMH-administered rats of short-term preclinical model of colon cancer led to the decrease in the expression of colonic proteins (cyclin B1, mdm2, cdc2) that control cell cycle as well as early response genes (c-fos, c-jun, c-myc) maintaining proliferation. Moreover, p-CA induced apoptosis via modulation of Bax/Bcl-2 ratio and improvement of detoxification potential [86]. Remarkably, the study evaluating reversal effects of bound polyphenol of inner shell (BPIS) from foxtail millet bran revealed that the fraction of molecular weight (MW) < 200 of BPIS, with FA and p-CA as main components, reversed multidrug resistance in HCT-8/Fu cells. The mechanism of its action was mediated via inhibition of proliferation, apoptosis induction, increase in accumulation of Rh-123 and decrease in the expression of multidrug resistance protein (MRP-1), p-glycoprotein (P-gp), and breast cancer resistance protein (BCRP) [87]. 

#### 2.2.4. Sinapic Acid

Sinapic acid (4-hydroxy-3,5-dimethoxycinnamic acid, SA) existing as free or esterified form is found in cereal grains, rye, wheat triticale, barley, oat, rye, rice, rapeseed, kale, white cabbage, turnip, broccoli, citrus fruits, and various herbs such as sage and thyme [2]. It exerts an antioxidant potential by acting as a free radical scavenging agent and increasing the activities of enzymatic and non-enzymatic antioxidants including SOD, CAT, and GSH [21]. Dimethyl sulfoxide extract of *Dianthus carmelitarum* with SA as one of the two most abundant phenolic compounds exerted selective cytotoxic effect on human colon cancer WiDr cells when compared with normal colon cells. It was also associated with S phase cell cycle arrest and induction of apoptosis mediated via reduced MMP [88]. Interestingly, the production of many phenolic constituents was associated with marine-derived fungus *Penicillium brevicompactum* treated with nicotinamide and sodium butyrate. Nicotinamide treatment-based resulted in the isolation and identification of nine compounds including SyA and SA which exerted free radical scavenging as well as antiproliferative abilities against HepG2 cell line [89]. Regarding anticancer activities of pure SA in human prostate cancer cells, it was associated with increase in the expression of Bax, caspase-3, -8, FAS, tissue inhibitor of metalloproteinase (TIMP-1), cytochrome c, and cadherin (CDH) 1 in PC-3 cells as well as increase in expression of caspase-3, -7, cytochrome c and Bax in LNCaP cells. However, it significantly decreased the expression of MMP-9 in PC-3 cells, while a decrease in the expressions of CDH2, MMP-2 and MMP-9 was observed in LNCaP [90]. Additionally, antiproliferative and HDAC inhibitory abilities of peanut phenolics including p-CA, FA, and SA were evaluated in MCF-7 and HeLa cells. Importantly, all of the compounds led to the dose-dependent inhibition of proliferation, concentration-dependent induction of apoptosis and indication of HDAC inhibition in both cell lines. Moreover, all of the mentioned compounds induced cell cycle arrest at G0/G1 phase in MCF-7 cells and S-phase arrest in HeLa cells induced by p-CA and FA [91]. 

## 3. Use of Phenolics in Clinical Research

The use of phenolics in clinical studies is not sufficiently represented in cancer research. Actually, we found hardly any association between phenolic acids and clinical cancer research. Regarding phenolic acids, the association between coffee intake and risk of breast cancer was evaluated in NIH-AARP Diet and Health Cohort Study conducted on 198 404 women. Despite that both of the main coffee constituents, caffeine and CA, were associated with suppression of mammary carcinogenesis in vivo and DNA methylation inhibition in vitro, no association between coffee intake and breast cancer was found in this large mostly postmenopausal prospective cohort [92]. Conceivably, topical antioxidant mixture of vitamin C, FA, and phloretin protected human skin against harmful UV irradiation, which suggests the complementary and synergistic effects of this mixture with sunscreen in human skin photoprotection [93]. Particularly, chemical analysis of black tea revealed high concentrations of GA [94]. Importantly, GA metabolites including 3MOGA were increased after consumption of five cups of black tea daily [95]. Actually, 3OMGA exhibits strong antiproliferative activity and the authors of the following study expected contribution of colonic phenolic breakdown products with tea health benefit in the digestive system. Therefore, a clinical trial evaluating an association between concentration of phenolic acids in plasma and urine of men consuming green or black tea and chemo-preventive abilities for colon cancer revealed that black tea-specific marker 3OMGA was increased after consumption of six cups of black tea when compared with water control [94]. 

## 4. Conclusions and Future Aspects

**Figure 4:**
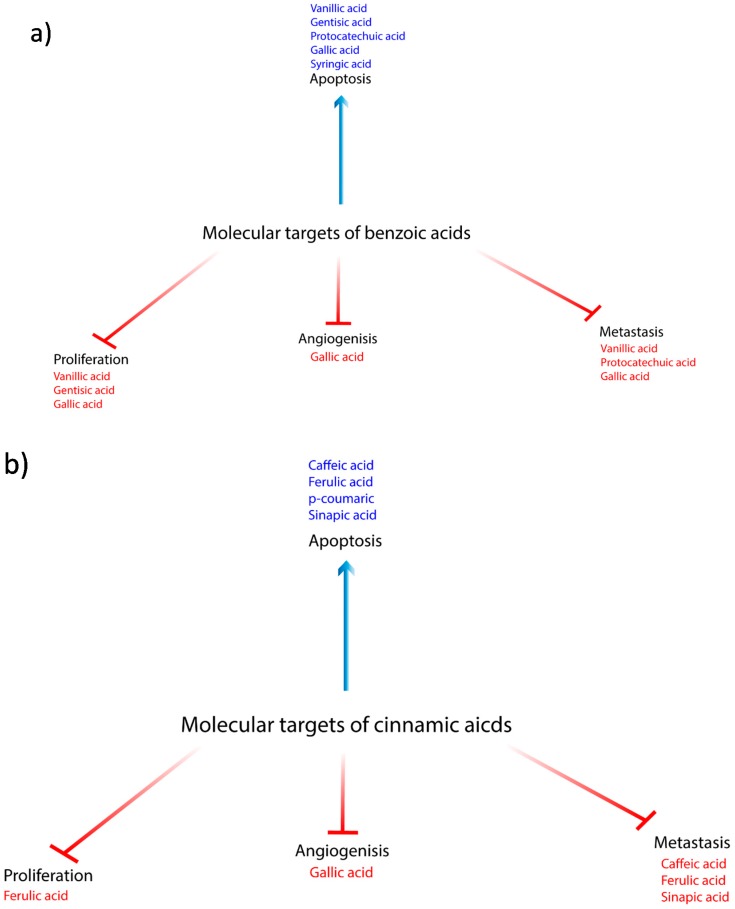
(**a**) Molecular targets of benzoic acids; vanillic, gallic, gentisic, protocatechuic and syringic in cancer treatment (**b**) Molecular targets of cinnamic acids; caffeic, ferulic, *p*-coumaric, and sinapic in cancer treatment.

Phytochemicals are currently gaining great importance in both cancer prevention and treatment due to their antioxidant, antiproliferative, antiangiogenic, proapoptotic, and anticancer properties. The role of benzoic and cinnamic acids is summarized in Figure 4a,b, respectively, and Table 1 [96,97]. Therefore, phenolics are robust candidates in the treatment of various types of cancer. Above all, the significant antineoplastic potential of benzoic and cinnamic acids as isolated plant phytochemicals is relatively well studied in in vitro as well as in vivo preclinical cancer research studies involving diverse cancer types. The lack of preclinical research focusing on the therapeutic effectiveness of VA against cancer may be partially explained/compensated by the occurrence of evidence demonstrating VA as potent chemo-preventive agents which was demonstrated in carcinogen-induced animal models of carcinogenesis [10,11]. Actually, the anti-cancer effectiveness of selected phytochemicals can be supported by an amount of evidence suggesting an important role of whole plant foods. Due to the presence of mixture of phytochemicals, whole plant food is suggested to exert better anti-cancer abilities when compared with isolated phytochemicals [96,97,98]. Therapeutic potential of whole plant food was demonstrated by several studies including evaluation of administration of *Vaccinium myrtillus* extract with GeA as the most abundant polyphenol [16], as well as RVSE with identified presence of GA [28]. Additionally, positive results were related to various whole plants characterized by presence of SyA, CA, and p-CA including *Menyanthes trifoliate* root extracts [49]. Extracts of *Anchusa azurea* Mill [66], *Rosa canina* extract [82], or extract of *Dianthus carmelitarum* [88]. Unlike clutivars of common beans [99], black raspberries did not inhibit the growth of cancer cells, despite that PCA was detected in rat prostates [100]. Importantly, several above-mentioned phytochemicals exerted also an ability to improve effectiveness or reduce side effects of conventional anticancer therapy. GeA as well as GA attenuated side effects of doxorubicin [17] and paclitaxel [43]. Additionally, GA was found to sensitize paclitaxel resistant cancer cells [44] and CA increased therapeutic potential of cisplatin [67]. Moreover, we emphasize important role of phenolics as epigenetic regulators. GA was demonstrated to be a potent inhibitor of DNMT as well as HDAC [38]. Furthermore, an ability of CA to attenuate cancer stem cells-like properties was mediated by microRNA-148a [59]. Also peanut phenolics including p-CA, FA, and SA were indicated to inhibit HDAC [91]. Above all, natural products represent potential anti-cancer agents. However, the bioavailability and efficient use of natural compounds is limited by their physico-chemical properties [26]. The use of nanomedicine in the area of drug delivery is associated with improvements in therapy efficacy, reduction of side effects, and prolonged bioavailability. GO-PEG-PCA-FA nanocarrier system designed for the PCA was found to be more effective anticancer agent when compared with free PCA [25]. Similar results were obtained also in case of PCA-ZnAL [26]. As well as in Fe₃O₄-PEG-GA [46] and PLGA-CS-PEG used to encapsulate GA [47]. Encapsulation of FE into cyclodextrin nanosponges led to improved solubility and cytotoxicity [80]. Eventually, the potential of natural compounds in association with benefit of nanocomposites need to be further studied [26].

In conclusion phenolics are robust candidates in the treatment of various types of cancer, they act on various molecular targets, (proliferation, angiogenesis, growth and differentiation, metastasis and apoptosis). However, additional studies in vitro and in vivo are required to ensure their efficacy and asses side effects. Due to the obvious lack of clinical intervention, we consider it necessary to implement evaluation of anticancer potential of phytochemicals, either isolated or mixtures, also in clinical practice.

## Figures and Tables

**Table 1:** Main anticancer pathways of phenolic acids.

Compound	Source	Anticancer Effect	Cancer Type	Type of Study	Mechanism	References
Vanillic Acid	*Angelica sinensis* and green tea	(-) growth and proliferation	Colon	in vitro	(-) mTOR/p70S6K/4E-BP1	[8]
Vanillic Acid		(+) apoptosis and antioxidant	Endometrial rat model	in vivo	(+) SOD, CAT, GPx, GSH, and vitamins C and E, (-) TBARS, LOOH	[13]
Vanillic Acid		(-) metastasis	Endometrial rat model	in vivo	(-) Cyclin D1, MMP -2, -9	[13]
Gentisic acid	citric fruits, grapes, artichoke, sesame, and olives	(+) apoptosis and antioxidant	Glioblastoma	in vitro	direct free radical scavenging activity indirect agonist of NRF2	[14]
Protocatechuic acid	plum, star anise, melissa, rosemary, cinnamon, sudan mallow, St. John’s wort, berries, cauliflower, and lentils	(+) apoptosis and antioxidant	Leukemia Gastric	in vitro	(+) ROS, DNA fragmentation, Bax, RB phosphorylation, Fas/FasL pathway, (-) Bcl-2, loss of mitochondrial membrane potential	[19]
Protocatechuic acid		(-) metastasis	Gastric	in vitro	(-) MMP-2	[21]
Gallic acid	chestnut green chicory, blackberry, raspberry, walnuts, chocolate, wine, green tea, and vinegar	(-) proliferation	Mesothelioma	in vitro	(-) VEGF and EGFR	[32]
Gallic acid		(+) apoptosis and antioxidant	Cervical Prostate Colon GBM	in vitro	(+) ROS & GSH (-) p38 MAPK Changes in calcium ion homeostasis	[27,30,34]
Gallic acid		(-) metastasis	Prostate Nasopharyngeal	in vitro	(-) MMP-1, -2, -9	[27,31,40]
Syringic acid	dates, olives, pumpkin, grapes, spices, acai, red wine, palm and honey	(+) apoptosis and antioxidant	Colon	in vitro	extrinsic, intrinsic, and mitochondrial pathways; (+) p53, Bax, Bak, Bad, Bid, Bim, Apaf1, AIF Smac, caspases-2, 3, 6, 7, 8 and 9, endoplasmic stress markers. cytochrome c, ROS (-) in the mitochondrial membrane potential, Bcl-2	[50,54]
Syringic acid		(+) apoptosis and antioxidant	Hamster buccal pouch	in vivo	(-) TBARS, LOOH, (+) enzymatic (SOD, CAT and Gpx) and non-enzymatic (vitamin E and GSH) antioxidants	[53]
Syringic acid		cell cycle	Colon	in vitro	arrest at S-phase, (-) cell cycle proteins CDK4, 6 and cyclins B, C, E1, H and (+) p19, p21^Cip1/Waf1^ and p27^kip1^	[54]
Caffeic acid	wheat, quinoa, triticale, barley, corn, oat, rye, rice, thyme, oregano millet, sage, and sorghum	antioxidant	Colon	in vitro	iron- chelating property (-) Fenton-induced oxidative damage and preventing the formation of free hydroxyl radicals	[55]
Caffeic acid		(-) metastasis	Lung Colon	in vitro	(-) cell adhesion	[3,57]
Ferulic acid	wheat, buckwheat, rice, corn, oats, rye, orange, corn, herbs, spices, sorghum, millet, quinoa, and barley	(-) metastasis	Endothelial Breast	in vitro	(-) FGF, cell adhesion, MMP -2, -9	[71,75]
Ferulic acid		Cell cycle arrest	Lung Colon Osteosarcoma	in vitro	G0/G1 arrest (-) CDK 2, 4 and 6, PI3K/Akt, Cyclins D1 and E	[3,57,73,74,77]
Ferulic acid		(-) proliferation	Breast	in vitro	(-) EGF	[78]
Ferulic acid		(+) apoptosis and antioxidant	Thyroid Lung Osteosarcoma	in vitro	(+) Bax, PARP, PUMA, NOXA, Bid, p53, PTEN, caspases-3 and -9, (-) CDK 4/6, CD 1, Bcl-2	[73,74,76,77]
*p*-Coumaric	wheat, barley oat, corn, rye, quinoa, rice, millet, honey sorghum barley grains and buckwheat	(+) apoptosis and antioxidant	Lung Prostate Colon	in vitro	(+) the ROS levels, Bax/Bcl-2 ratio, loss of mitochondrial membrane potential, Rh-123(-) MRP1, P-gp, and BCRP	[82,85,86,87]
*p*-Coumaric		anti-inflammatory	Colon	in vitro	(-) IL-6, COX-2, TNF-α, PGE2, p-p65 and p-IκBα	[84]
Sinapic acid	cereal grains, rye, wheat triticale, barley, oat, rye, rice, rapeseed, kale, white cabbage, turnip, broccoli, citrus fruits, sage and thyme	(+) apoptosis and antioxidant	Prostate	in vitro	(+) activities of enzymatic and non- enzymatic antioxidants; SOD, CAT, and GSH (+) Bax, caspases -3, -7, -8, FAS, TIMP-1, cytochrome c	[21,90]
Sinapic acid		(-) metastasis	Prostate	in vitro	(-) MMP-2, -9, CDH 1, 2	[90]

## Abbreviations

Apoptosis Inducing Factor	AIF
Apoptosis Protease Activating Factor-1	Apaf-1
B-Cell Lymphoma 2	Bcl-2
Bcl-2 Antagonist and Killer	Bak
Bcl-2 Associated Death Promoter	Bad
Bcl2-Associated X Protein	Bax
Bcl-2-Like Protein 11	Bim
BH3 Interacting-Domain Death Agonist	Bid
Breast cancer resistance protein	BCRP
Catalase	CAT
Cadherin	CDH
C-Jun N-Terminal Kinase	JNK
Cyclin Dependent Kinases	CDKs
Epidermal Growth Factor Receptor	EGFR
Extracellular matrix	ECM
Extracellular signal-regulated kinase	ERK
Glutathione	GSH
Glutathione Peroxidase	GPx
Hypoxia Inducing Factor	HIF-1
Lipid Hydroperoxides	LOOH
Macrophage Inhibitory Cytokine	MIC-1
Matrix Metalloproteinases	MMP
Mechanistic target of rapamycin	mTOR
Mitogen-activated protein kinase	MEK
Mitogen-Activated Protein Kinase	MAPK
Multidrug resistance protein	MRP
Nicotinamide Adenine Dinucleotide Phosphate Hydrogen	NADPH
Nicotinamide Adenine Dinucleotide Phosphate Oxidases	Nox
N-methyl-N’-nitro-N-nitrosoguanidine	MNNG
Nuclear Factor Erythroid 2-Related Factor 2	NRF2
Nuclear factor-kappa B	NF- κB
Organic Anion Transporter-3	OAT3
P-glycoprotein	P-gp
Phosphoinositide 3-Kinase	PI3K
Poly (ADP-Ribose) Polymerase	PARP
Protein Kinase B	Akt
Protein Tyrosine Phosphatase Receptor	PTP-κ
Reactive Oxygen Species	ROS
Rhodamine-123 Second Mitochondrial Derived Activator of Caspase	Rh-123 Smac
Superoxide Dismutase	SOD
Tissue inhibitor of metalloproteinase	TIMP-1
Thiobarbituric Acid Reactive Substances	TBARS
Vascular Endothelial Growth Factor	VEGF
